# Iterative pruning PCA improves resolution of highly structured populations

**DOI:** 10.1186/1471-2105-10-382

**Published:** 2009-11-23

**Authors:** Apichart Intarapanich, Philip J Shaw, Anunchai Assawamakin, Pongsakorn Wangkumhang, Chumpol Ngamphiw, Kridsadakorn Chaichoompu, Jittima Piriyapongsa, Sissades Tongsima

**Affiliations:** 1 NECTEC 112 Thailand Science Park, Paholyothin Road, Klong 1, Klong Luang, Pathumtani 12120, Thailand; 2 BIOTEC 113 Thailand Science Park, Paholyothin Road, Klong 1, Klong Luang, Pathumtani 12120, Thailand; 3 Division of Molecular Genetics, Department of Research and Development, Faculty of Medicine Siriraj Hospital, Bangkok 10700, Thailand

## Abstract

**Background:**

Non-random patterns of genetic variation exist among individuals in a population owing to a variety of evolutionary factors. Therefore, populations are structured into genetically distinct subpopulations. As genotypic datasets become ever larger, it is increasingly difficult to correctly estimate the number of subpopulations and assign individuals to them. The computationally efficient non-parametric, chiefly Principal Components Analysis (PCA)-based methods are thus becoming increasingly relied upon for population structure analysis. Current PCA-based methods can accurately detect structure; however, the accuracy in resolving subpopulations and assigning individuals to them is wanting. When subpopulations are closely related to one another, they overlap in PCA space and appear as a conglomerate. This problem is exacerbated when some subpopulations in the dataset are genetically far removed from others. We propose a novel PCA-based framework which addresses this shortcoming.

**Results:**

A novel population structure analysis algorithm called iterative pruning PCA (ipPCA) was developed which assigns individuals to subpopulations and infers the total number of subpopulations present. Genotypic data from simulated and real population datasets with different degrees of structure were analyzed. For datasets with simple structures, the subpopulation assignments of individuals made by ipPCA were largely consistent with the STRUCTURE, BAPS and AWclust algorithms. On the other hand, highly structured populations containing many closely related subpopulations could be accurately resolved only by ipPCA, and not by other methods.

**Conclusion:**

The algorithm is computationally efficient and not constrained by the dataset complexity. This systematic subpopulation assignment approach removes the need for prior population labels, which could be advantageous when cryptic stratification is encountered in datasets containing individuals otherwise assumed to belong to a homogenous population.

## Background

Allele frequencies vary across populations because of differences in ancestry; these differences arise from many factors such as migration, selection and drift. Hence, populations are genetically substructured. The information obtained from resolving population substructure can be used to infer population history. Furthermore, human disease association studies must account and correct for the population substructure to reduce spurious associations and reveal the predisposing factors of disease [1]. Analysis of population stratification must meet four main challenges namely: (i) detecting structure, (ii) assigning individuals to subpopulations, (iii) determining the number of optimal, or primal, subpopulations (*K*) and (iv) determining the proportions of ancestral subpopulations (admixture) [2].

With the advent of high throughput genotyping, increasingly large genotypic datasets (e.g. HapMap dataset of 3.5 million single nucleotide polymorphism (SNP) arrays from 270 individuals[3]) will provide progressively difficult challenges for population structure analysis. Therefore, to keep abreast with the ever increasing size and complexity of genotypic data, refinement of existing analytical methods and entirely novel approaches will be needed to resolve subpopulations. Several different methods have been proposed to address some aspects of the population substructure problem. These methods can be categorized into two main approaches, namely parametric and non-parametric-based. STRUCTURE is the "gold standard" parametric-based algorithm for population stratification analysis [2,4] because it addresses all four substructure analysis challenges. However, STRUCTURE imposes a high computational burden and it is thus impractical for the large datasets typically analyzed nowadays. STRUCTURE was originally designed to be used with microsatellite markers, which are generally more informative than SNPs. Nonetheless, it is generally accepted that the computational burden of analyzing large SNP datasets with STRUCTURE is a trade-off, since large numbers of SNP markers can be easily typed in parallel on array platforms, in contrast to the laborious electrophoretic methods for typing microsatellites. STRUCTURE also has other weaknesses, mainly its inference of *K*, which requires extensive statistical testing of several STRUCTURE runs performed with increasing *K*. STRUCTURE's inference of *K *can also vary according to the model used [4], hence the determination of admixture, which is highly dependent on the *K *value used needs to be interpreted with caution. Alternative parametric methods, such as L-POP [5], PSMIX [6], *frappe *[7], BAPS [8,9], and TESS [10] are more computationally efficient. These algorithms are used mainly to infer ancestry (admixture) using statistical inference methods.

For non-parametric methods, the algorithms in this class do not require model assumptions. Principal Components Analysis (PCA) is the most widely used method for visualizing structure which uses a covariance matrix for eigenanalysis, allowing representation of individuals as data points in scatter-plots. Principal Coordinate Analysis (PCoA) is an alternative method for eigenanalysis which uses an allele-sharing distance (ASD) matrix, and gives different scatter-plot patterns from PCA [11,12]. Note that PCA and PCoA are not methods for assigning individuals nor estimating *K *and are often used merely to visualize the population structure trend.

The most popular PCA-based algorithm applied to population structure analysis is EIGENSTRAT/SmartPCA [13,14], which has been used by several investigators for large datasets typically required for studies of human population structure and disease association [15-17]. This algorithm employs a computationally-efficient variant of eigenanalysis to report the probability of population substructure according to Tracy-Widom (TW) distribution.

In a typical population dataset, genetic distances vary among subpopulations and PCA scatter-plots can reveal the most genetically isolated subpopulations as distinct clusters of individuals in a small number of principal components. Hence, supervised clustered with prior assumption of the number of *K *subpopulation clusters can be done to assign individuals. Conversely, closely related subpopulations will occupy a confined feature space and appear as a conglomerate. For example, in the scatter-plot of PC1 versus PC2, conglomerates containing individuals with different population labels are frequently observed. In some cases, the distinction between closely related subpopulations is apparent in a greater number of principal components [18]. Thus, in order to resolve closely related subpopulations, individuals must be separated using a clustering algorithm working in multidimensional PCA space [19-21]. Clustering algorithms require separation in axes of variation. However, clusters in some axes may merge into a single cluster, and hence clustering algorithms can become confused when too many axes of variation are used [22]. In general, the informative axes of variation are contained within the rank of matrix [23]. Therefore, the number of principal components should be optimized and not exceed a certain number for each dataset.

One way to improve the resolution of closely-related subpopulations in PCA scatter-plots is by removing genetically distant individuals from the dataset. In the investigation of European human genetic substructure using 300K SNP arrays [24], it was found that prior exclusion of individuals belonging to certain groups improved substructure resolution of the other groups, e.g. removal of Ashkenazi Jewish individuals led to clearer substructuring of other northern European groups. This data "cleaning" approach is clearly advantageous, although the method as described in [24] is *ad hoc *and unable to detect and remove subtle outliers [25]. Clearly, this approach is not feasible for datasets composed of individuals presumed to belong to a homogenous population without any distinguishing labels, for instance disease association studies carefully controlled for ethnicity and geographical origin. With sufficient number of markers and individuals, cryptic structure in an apparently homogenous population can be detected using PCA [13,14], which cannot be resolved by current unsupervised clustering methods, with no assumptions of *K *[21].

To determine the primal *K *for unsupervised clustering, the Gap statistic [26] is employed on the AWclust results [19] and Density-Based Means clustering results [21]. Alternatively, the Bayesian Information Criterion (BIC) approach for determining *K *can be applied to the clustering results [20]. Calculating the Gap statistic and BIC are computationally intensive, thus these approaches are impractical for highly structured datasets. Furthermore, these approaches are sensitive to noisy non-informative PCs, which may explain why they are not appropriate for highly structured datasets with a large *K*. All unsupervised clustering approaches currently applied to PCA do not assign individuals into subpopulations according to genetic distance between subpopulations in a fully comprehensive and systematic fashion. These algorithms thus have insufficient discriminatory power to assign individuals to subpopulations and determine *K *for highly structured datasets.

In this study, we propose a non-parametric analytical framework which incorporates several key refinements of the PCA-based approach. The new algorithm, which we call iterative pruning PCA (ipPCA) addresses three of the main challenges for population structure analysis, namely detection of structure, assigning individuals to subpopulations and determining the primal *K *with greater accuracy than previously proposed algorithms. The ipPCA algorithm utilizes a novel unsupervised clustering heuristic which markedly improves resolution of population substructure by an iterative process. In this method, groups of individuals are systematically bisected according to genetic similarity at each step, continuing until a termination point (defined by a test statistic) is reached revealing the underlying subpopulations.

## Results

### Algorithm

The ipPCA algorithm utilizes non-redundant principal components to construct a transformed domain of the input data, which can be mapped to PCA space. By means of selecting a limited number of principal components, dimension reduction can be achieved. The PCA domain allows each input individual to be represented as a datum point in a scatter-plot, or in clustering analysis. The ipPCA technique constructs the transformed domain based on a covariance matrix of the data matrix containing SNP genotypic data encoded as 0, 1 or 2 elements.

where *M *and *N *are the number of markers and individuals respectively. Note that *M *is normally much larger than *N*.

Since the covariance matrix is usually a large square matrix, it is memory and computationally intensive to compute the PCA space. However, there is an alternative technique to compute the transformed domain with less computational burden [23]. Instead of using the data covariance matrix, the PCA space can be constructed by decomposing to a modified data matrix *XX*^*T*^, which is much smaller than the covariance matrix. For example, a dataset with 200 individuals and 1000000 markers would have 1000000 × 1000000 elements in the covariance matrix compared to 200 × 200 elements in the *XX*^*T *^matrix. The modified data matrix can be factored as *USV*^*T *^= *XX*^*T *^where

Then, the eigenvectors can be computed by

where *k *is the rank of the data matrix and  are the eigenvectors. Then, the PCA space is constructed based on these eigenvectors.

Because ipPCA is sensitive to the data pattern, data quality is crucial for accuracy of the algorithm. Typically, it is necessary to clean the data before performing ipPCA. Technical limitations in genotyping occasionally lead to missing values at some loci. To check whether missing data by itself can create substructure, all missing data are encoded as zero and other loci are encoded as one. The 0-1 encoded dataset is analyzed by SmartPCA [13]. If substructure can be observed as clear outlier individuals on the periphery of the two PC scatter-plot, these individuals can be removed from the dataset, as suggested by [13].

Before performing ipPCA, a quality control check is performed on the 0-1-2 encoded data matrix. In this case, frequency counts are computed on each locus to ensure that entries encoded as zero are homozygous major allele (wild-type), entries encoded as one are heterozygous and entries encoded as two are homozygous minor allele. The pre-processing steps and the ipPCA framework can be summarized as follows:

### Pre-processing steps

Check if missing values in the input dataset cause detectable substructure using EIGENSTRAT/SmartPCA and if significant substructure is reported, remove individuals with missing data which cause substructure (identified as outliers on PC1-PC2 scatter-plot).

### ipPCA steps

1. Make matrix *X*_*i *_for each data group, i.e. dataset or nested dataset which contains substructure.

2. Use singular value decomposition (SVD) [23] to factorize the  data into 

3. Project all individuals into PC space using the number of PCs equal to the data matrix rank [18].

4. Check terminating condition on the *S*_*i *_using the TW test statistic implemented in EIGENSTRAT/SmartPCA [13]. The default TW *p*-value threshold used for detectable structure is conservative (*p *> 10^-12^) and lies well above the Baik Ben Arous Péché (BBP) threshold for significance among empirically tested datasets (see [13] for details).

(a) Terminate if TW test statistic is insignificant for the first PC (*p *> 10^-12^) (subpopulation resolved)

(b) Otherwise, proceed to the next step

5. Apply fuzzy *c-*means [27] to separate individuals *X*_*i *_into two clusters.

6. Repeat from step 1 until all the data are terminated.

When all the iterative processes are terminated, the number of subpopulations can be determined by counting all the terminal nodes (determination of *K*). Note that replicate ipPCA runs are typically performed to test the robustness of the clustering algorithm, which could affect the assignment accuracy and determination of *K*. For datasets with simple structures, replicate runs may be unnecessary; however, the extra computational effort of performing replicate runs is minimal.

### Testing

The power and robustness of the proposed ipPCA algorithm was explored and optimized using simulated datasets. The performance of ipPCA was then tested on three real datasets, namely HapMap, bovine, and Shriver's datasets. Finally, we compared the results of our algorithm with the results from existing tools: STRUCTURE, BAPS and AWclust. STRUCTURE version 2.2 was downloaded from J. Pritchard's website [28] and applied using the following parameters: 100000 burn-ins, 100000 runs, admixture model, no LD model. Individuals were assigned to subpopulations according to the highest reported probability value. For BAPS, the software was downloaded from [29] and executed using the mixture model. For AWClust analysis, AWClust was downloaded from [30] and the algorithm's default parameters were selected.

### Iterative pruning PCA clustering: a way to improve discriminatory power

**Figure 1:**
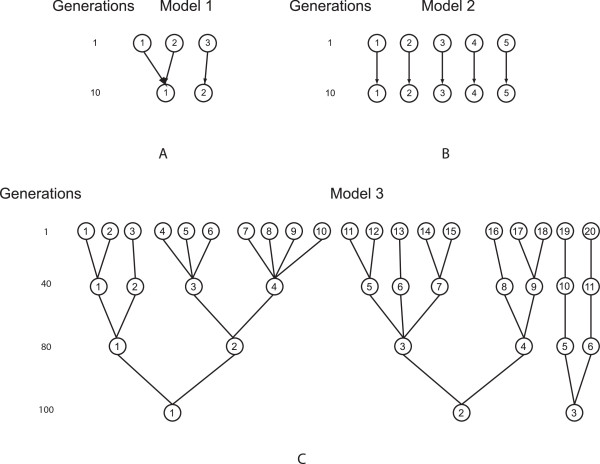
**Population history trees for generating simulated datasets**. The GENOME tool [31] was used to generate the simulated datasets. A) three subpopulations mixed model (model 1) B) five independent subpopulations (model 2) C) twenty subpopulations (model 3)

The program GENOME [31] was employed to generate simulated genotypic data under the Wright-Fisher neutral coalescent model (backward in time) [32]. Three evolutionary models (called population histories in GENOME) were constructed (Figure 1). The model 1 dataset (Figure 1A) contains three subpopulations derived from two ancestral populations. The model 2 dataset (Figure 1B) contains five independent subpopulations with no admixture. The model 3 datasets (Figure 1C) contain 20 subpopulations derived from three ancestral populations. Parameter settings used to generate each model are as follows:

The model 1 dataset parameters:

-pop 3 50 50 50 -c 20 -s 500 -N model1.txt

The model 2 dataset parameters:

-pop 5 50 50 50 50 50 -c 20 -s 500 -N model2.txt

The model 3 dataset parameters:

-pop 20 50 50 50 50 50 50 50 50 50 50 50 50 50 50 50 50 50 50 50 50 -c 20 -s 500 -N model3.txt

Using the above parameters, we generated 10000 SNPs for each simulated dataset. Note that the model 3 population history was used to generate 30 datasets to test the robustness of ipPCA. All simulated datasets and the population history files (model1.txt, model2.txt, and model3.txt) analyzed in this study can be downloaded from http://www4a.biotec.or.th/GI/tools/ippca

**Figure 2:**
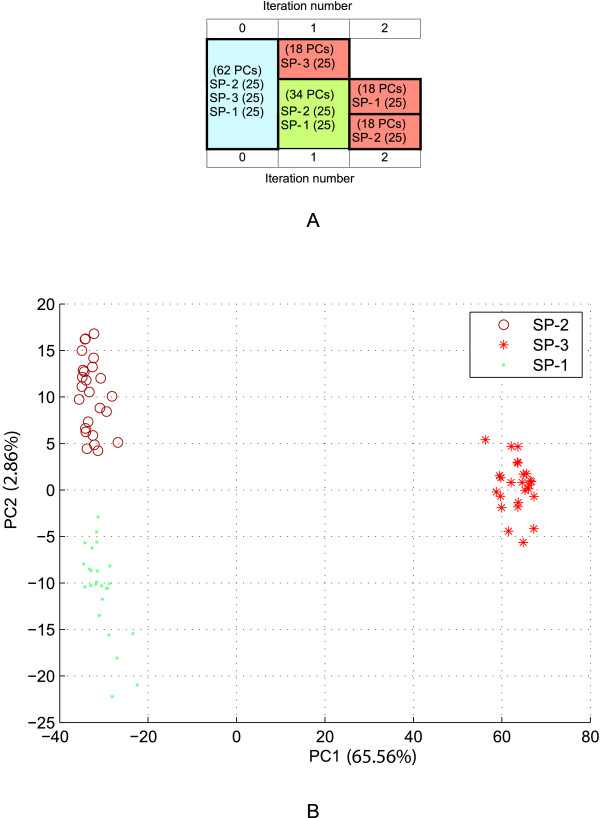
**ipPCA analysis of simulated data model 1 with 3 subpopulations**. A) Consensus subpopulation tree from ten ipPCA replicates. Each cell contains labels SP-1, SP-2 or SP-3, which refer to subpopulation labels used in simulation. The number of individuals is shown in parentheses next to each label. The number of PCs used for clustering is indicated in parentheses in each cell. The blue cell indicates the entire dataset. Nested datasets containing unresolved structure are in green, while the terminated red cells represent resolved subpopulations. B) Scatter-plot using the first and second principal components (PC1 vs. PC2) of the entire dataset (iteration 0 of ipPCA). Each datum point represents an individual. Each subpopulation label is denoted by a separate symbol (see inset). The variation captured by each PC is indicated in parenthesis next to the axis label.

**Figure 3:**
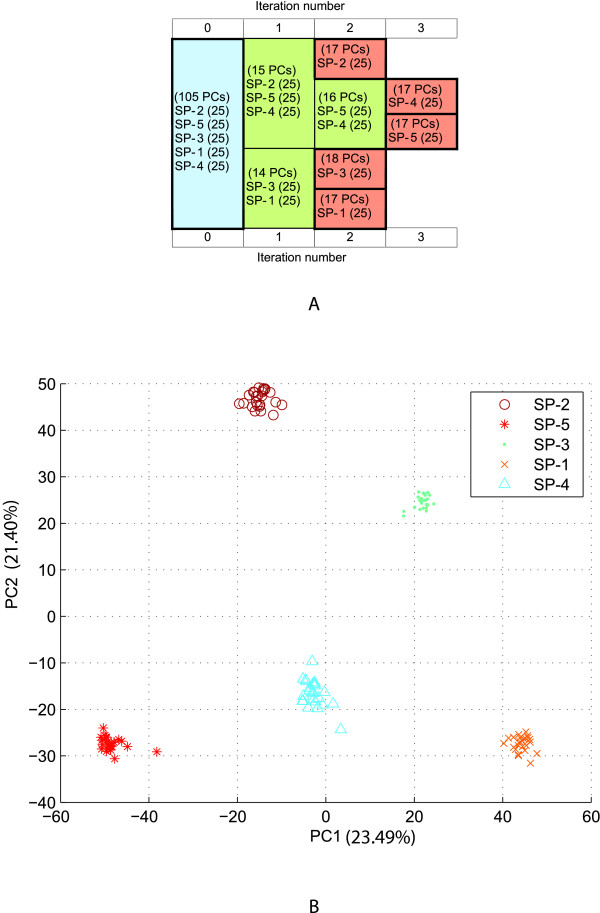
**ipPCA analysis of simulated data model 2 with 5 subpopulations**. A) Consensus subpopulation tree on ten replicated ipPCA runs. Each cell contains labels SP-1, SP-2, SP-3, SP-4 or SP-5, which refer to subpopulation labels used in simulation. The number of PCs used for clustering is indicated in parentheses in each cell. The blue cell indicates the entire dataset. Nested datasets containing unresolved structure are in green, while the terminated red cells represent resolved subpopulations. B) Scatter-plot using the first and second principal components (PC1 vs. PC2) of the entire dataset (iteration 0 of ipPCA). Each datum point represents an individual. Each subpopulation label is denoted by a separate symbol (see inset). The variation captured by each PC is indicated in parenthesis next to the axis label.

The performance and robustness of ipPCA to resolve structure were investigated using simulated datasets of increasing complexity. Two simple population structure models (*K *= 3 and 5 subpopulations) and one complex model (*K *= 20 subpopulations) were simulated using the GENOME tool [31]. For the first two simple models (models 1 and 2), ipPCA can resolve *K *consistent with the models and assign all individuals correctly to the corresponding subpopulations (Figures 2 and 3). For the highly complex model (model 3), 30 simulated datasets were generated using the same evolutionary model. The structure resolved by ipPCA was highly consistent with the model, for both number of inferred *K *and the individuals assigned to each subpopulation (mode *K *= 20, range 19-22; mean mis-assignment rate 6.07%, range 1.9%-17.5%), see additional file 1). To test the reproducibility of the clustering algorithm, ten replicates of ipPCA were performed on each data set. The results for each dataset were reproduced exactly, with no deviation in individual assignment or inference of *K *between replicates in each case (data not shown).

### Assignment of individuals to subpopulations in real datasets is reproducible and consistent with population labels

Three real datasets, namely HapMap, bovine, and Shriver's, were analyzed in this work. The HapMap dataset, retrieved from [33], comprises individuals with four population labels: Han Chinese from Beijing (CHB), Japanese from Tokyo (JPT), Caucasian European from Utah (CEU), and Yoruba from Ibadan (YRI) with 1533661 SNPs. Fifty thousand SNPs were uniformly re-sampled from this SNP pool [3]. Starting from the first marker, a moving window was used to select SNPs in an even spacing fashion, such that every 30^th ^marker was selected from the 1533661 markers (for full list of SNP markers used see additional file 2). Data pre-processing ipPCA was performed and two outlier JPT individuals were removed. The bovine dataset was downloaded from [34]. The bovine SNP data (9329 SNPs) are publicly available as part of the Bovine Genome Project [35]. After data pre-processing, no outliers were detected. Shriver's worldwide human SNP dataset of 307 individuals with 14 different ethnic/geographical labels was provided by Prof. Mark D. Shriver, which is a dataset expanded from the one originally published in [36]. This dataset consists of 11555 SNPs for each individual, evenly distributed over the entire genome. After data pre-processing, no outliers were detected.

**Figure 4:**
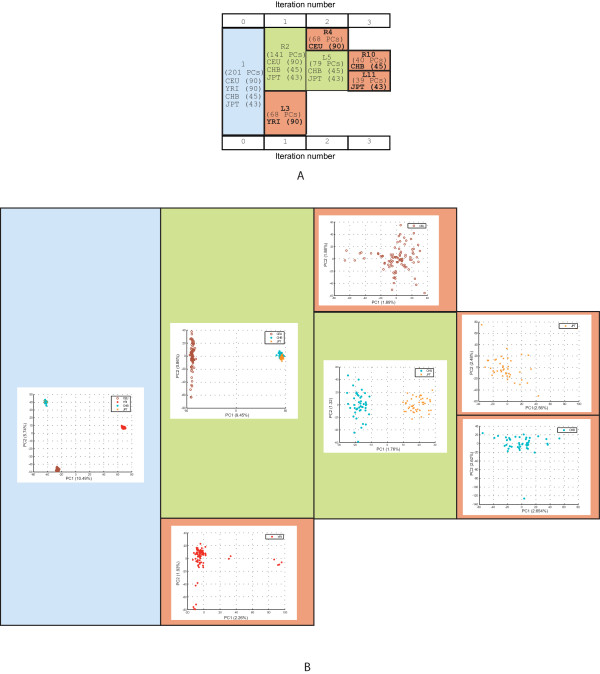
**ipPCA analysis of HapMap human dataset**. **A) Consensus subpopulation tree**. Each cell contains population labels YRI, CEU, CHB, or JPT. The number of individuals is presented in parentheses next to each label. The number of PCs used for clustering is indicated in parentheses in each cell. The blue cell indicates the pre-processed dataset. Nested datasets containing unresolved structure are in green, while the terminated red cells represent resolved subpopulations. **B)Scatter-plots using the first and second principal components (PC1 vs. PC2)**. Each datum point represents an individual. Each population label is denoted by a separate symbol (see inset). The blue frame contains a scatter-plot of ipPCA iteration 0. Scatter-plot of the nested dataset at iteration 1 is framed in green. Scatter-plots of resolved subpopulations are framed in red. The variation captured by each PC is indicated in parenthesis next to the axis label.

The HapMap dataset was analyzed by ipPCA, which contains individuals from four distinct geographical regions (YRI, CEU, CHB, and JPT). All individuals in each of the four subpopulations defined by ipPCA share the same distinguishing population label (Figure 4). The YRI subpopulation was defined in iteration 1 while the CEU was defined in iteration 2. Finally, the CHB and JPT subpopulations were defined in iteration 3. Exactly the same assignment results were obtained from ten replicates using 50000 SNPs and a single ipPCA run using the entire 1533661 SNP collection (data not shown).

The bovine dataset contains individuals from nine breeds considered to be genetically distinct subpopulations. The Brahman (BRM) breed cattle are zebu (also known as *B. indicus*), considered a sub-species very distinct from the taurine cattle breeds. Santa Gertrudis (SGT) is a composite breed derived from BRM and Shorthorn breed cattle. The other breeds, Angus (ANG), Charolais (CHL), Limousin (LMS), Hereford (HFD), Norwegian red (NRC), Jersey (JER) and Holstein (HOL) are taurine cattle and European in origin (for breed descriptions see [37]).

**Figure 5:**
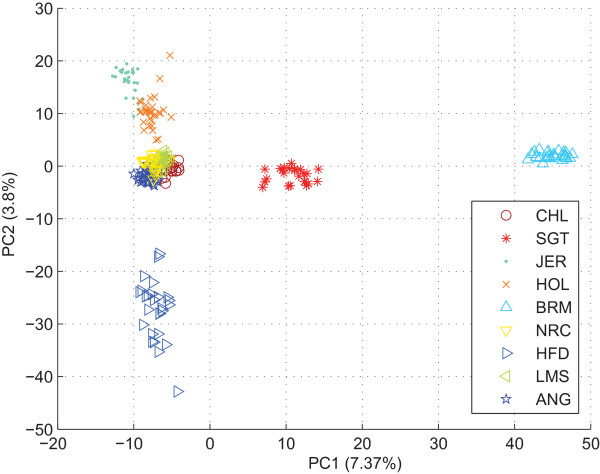
**The PCA scatter-plot of the entire bovine dataset for the zeroth iteration of ipPCA**. The plot was made using the first two principal components (PC1 vs. PC2). Each datum point represents an individual. Each subpopulation label is denoted by a separate symbol (see inset). The variation captured by each PC is indicated in parenthesis next to the axis label.

**Figure 6:**
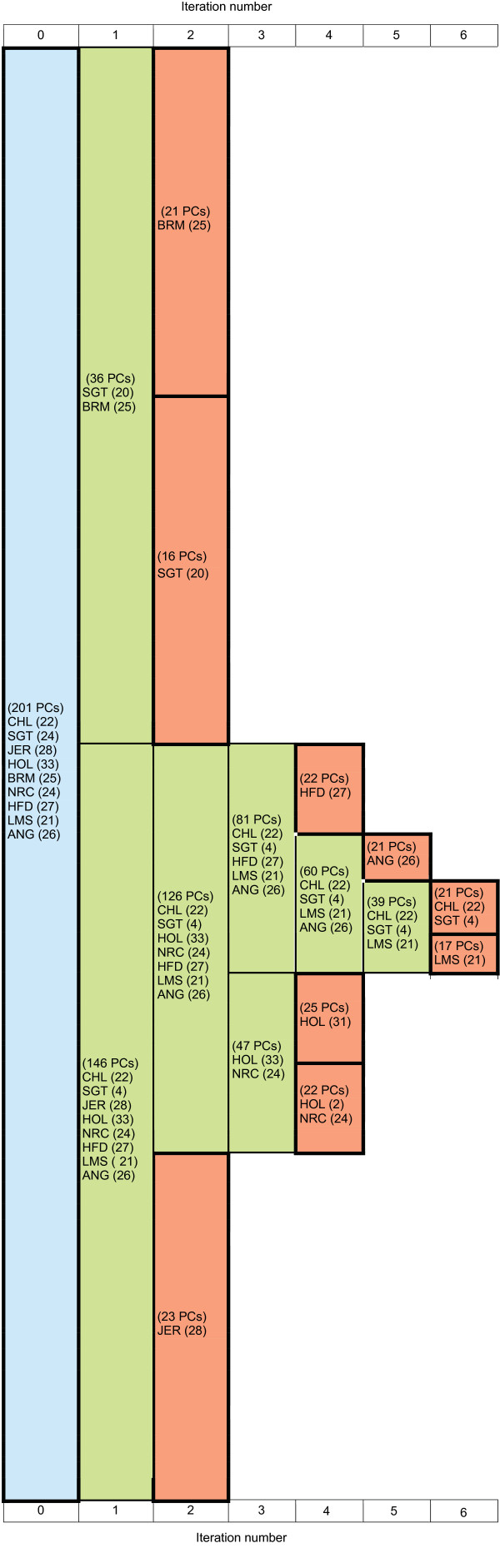
**Bovine consensus subpopulation tree on ten replicate ipPCA runs**. Each cell contains breed (population) labels CHL, SGT, JER, HOL, BRM, NRC, HFD, LMS, or ANG. The number of individuals is presented in parentheses next to each label. The number of PCs used for clustering is indicated in parentheses in each cell. The blue cell indicates the entire dataset. Nested datasets containing unresolved structure are in green, while the terminated red cells represent resolved subpopulations.

Scatter-plot analysis of the entire bovine dataset showed that individuals from the HFD, JER, HOL, LMS, ANG, CHL and NRC taurine breeds appear as a conglomerate and are separated from BRM and SGT individuals (Figure 5). Figure 6 shows the resulting consensus subpopulation tree from ten ipPCA replicates, which all gave the same results (data not shown). The ipPCA program required six iterations to define all subpopulations in the bovine dataset. After the second iteration, the BRM and SGT cluster was divided satisfying the termination criterion, thus defining two subpopulations composed of BRM and SGT individuals, respectively. Additionally, a third subpopulation composed of JER individuals was defined and separated from the taurine cluster. After more iterations of ipPCA, a further six subpopulations were defined in which each subpopulation is largely composed of individuals of the same breed.

**Figure 7:**
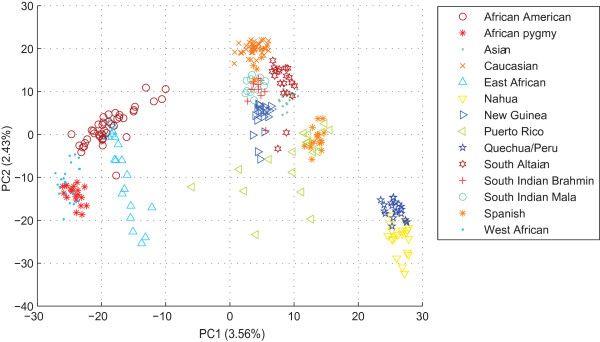
**The PCA scatter-plot of Shriver's entire dataset for the zeroth iteration of ipPCA**. The plot was made using the first two principal components (PC1 vs. PC2). Each datum point represents an individual. Each population label is denoted by a separate symbol (see inset). The variation captured by each PC is indicated in parenthesis next to the axis label.

**Figure 8:**
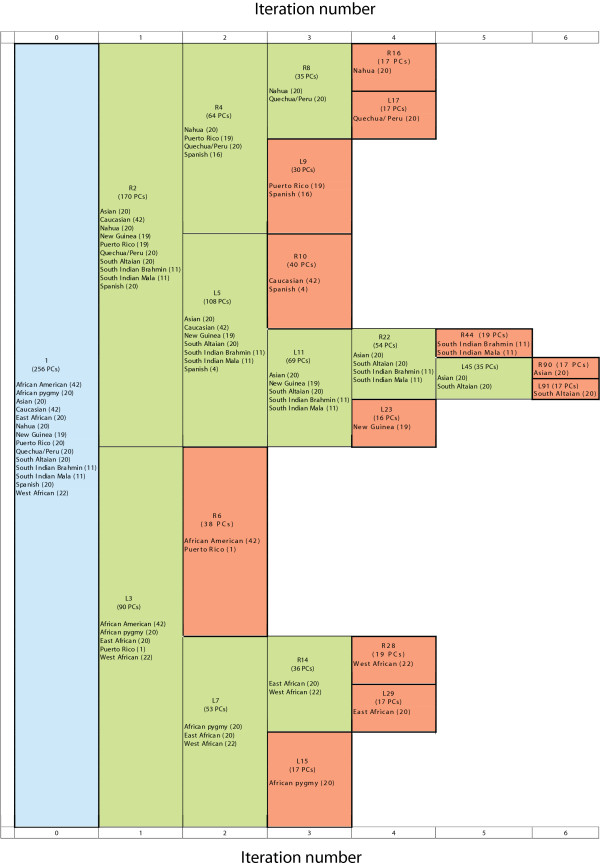
**Shriver's consensus subpopulation tree on ten replicate ipPCA runs**. Each cell contains population labels African American, African Pygmy, Asian, Caucasian, East African, Nahua, New Guinea, Puerto Rican, Quechua/Peru, South Altaian, South Indian Brahmin, South Indian Mala, Spanish, or West African. The number of individuals is presented in parentheses next to each label. The number of PCs used for clustering is indicated in parentheses in each cell. The blue cell indicates the entire dataset. Nested datasets containing unresolved structure are in green, while the terminated red cells represent resolved subpopulations.

Shriver's dataset was then analyzed by ipPCA. From the scatter-plot analysis of the entire dataset, it can be observed that individuals are broadly grouped according to their geographical origins with some overlap of individuals with different labels (Figure 7). The consensus ipPCA result showed that after the second iteration of ipPCA, a subpopulation composed of African Americans and a Puerto Rican individual was defined. After further iterations of ipPCA, more subpopulations were defined with each containing individuals with shared geographical and/or ethnic origins. After six iterations, twelve subpopulations were defined with no further substructure found (see Figure 8). Subpopulation assignments were robust. Only one ipPCA replicate differed from the consensus (see additional file 3).

The assignments of individuals to subpopulations and *K *inferred by ipPCA were compared with other algorithms. Table 1 shows comparison between different algorithms for inferring *K *from 35 datasets. For ipPCA, the inference of *K *was highly robust, with variation observed only among the most highly structured model 3 populations. There was no concordance between the algorithms for inference of *K*. Given the discordancy between the algorithms, we then investigated the individual assignments made by each algorithm.

**Table 1:** Comparison of four algorithms (AWclust, STRUCTURE, BAPS and ipPCA) for inferring the number of primal subpopulations (*K*).

Dataset	AWclust	STRUCTURE	BAPS	ipPCA
model 1 simulated (3 subpopulations)	3	3	2	3
model 2 simulated (5 subpopulations)	5	6	5	5
model 3 simulated (20 subpopulations, 30 datasets)	N/A^1^	N/D^2^	11^3^	20^3^
HapMap (4 population labels with 50000 SNPs)	3	3	3	4
bovine (9 population labels with 9329 SNPs)	N/A^1^	10	14	9
Shriver's (14 population labels with 11555 SNPs)	N/A^1^	14^4^	5	12

^1 ^Not applicable since AWclust gap statistics limits *K *< 8

^2 ^Not done owing to the computational constraint

^3 ^Modal value from 30 simulated datasets

^4 ^There are two subpopulations with no individuals assigned to them

**Figure 9:**
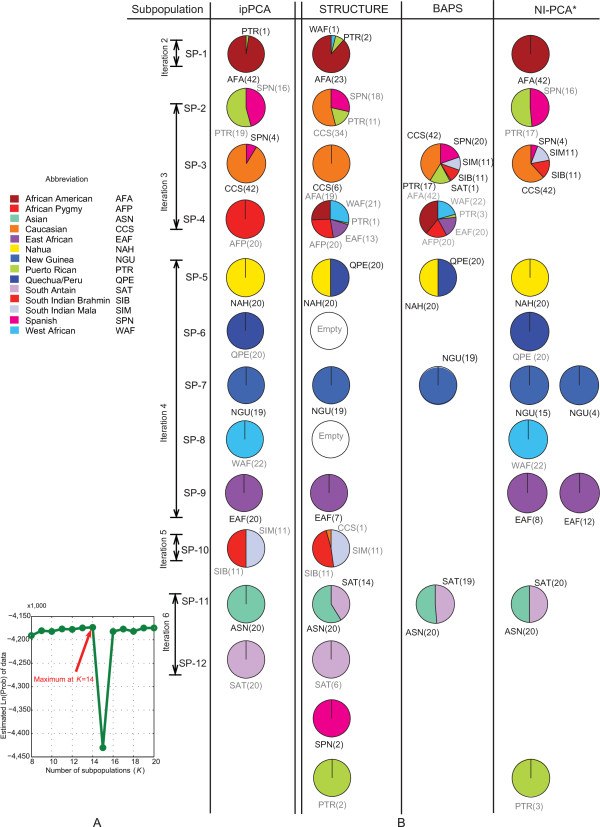
**Population assignment comparison of Shriver's dataset among ipPCA, STRUCTURE, BAPS, and non-iterative PCA clustering algorithms**. A) Log probability data plot of inferred *K *for STRUCTURE results (no admixture model) for *K *= 8 to *K *= 20; STRUCTURE parameters: 100000 simulation iterations for burn-ins and 100000 additional iterations for parameter estimation. B) Side-by-side comparison of subpopulation assignment by ipPCA, STRUCTURE, BAPS and non-iterative PCA clustering (NI-PCA). Population labels are the same as in [36].

For datasets of low complexity, assignment of individuals to subpopulations was broadly consistent among the STRUCTURE, BAPS, AWclust, and ipPCA algorithms (see additional file 4 for AWclust results, additional file 5 for STRUCTURE results, and additional file 6 for BAPS results). Given the greater discordance between algorithms for inference of *K *from highly structured datasets, a more detailed comparison of individual assignments to subpopulations was made between STRUCTURE, BAPS and ipPCA. The comparison was made using Shriver's highly structured dataset for the *K *reported by each algorithm (Figure 9). Each of the twelve subpopulations defined by ipPCA contain similar number of individuals (range 19-46), and the assignments of individuals to subpopulations SP-4, SP-5, SP-6, SP-7, SP-8, SP-9, SP-11 and SP-12 are consistent with the population labels. Four subpopulations, namely SP-1, SP-2, SP-3 and SP-10 contain individuals of more than one population label.

The subpopulations assigned by STRUCTURE and BAPS differed markedly to ipPCA in the number of individuals contained within each. For STRUCTURE, two subpopulations have no assigned individuals, five subpopulations have fewer than ten individuals and two subpopulations have more than 50 assigned individuals. For BAPS, there are two large subpopulations with more than 50 individuals and three smaller subpopulations. Inspection of the population labels of the assigned individuals shows that STRUCTURE and BAPS assigned a subpopulation containing individuals from three geographically disparate African locations together with African Americans and Puerto Ricans in contrast to ipPCA, which assigns African Pygmy, West African and East African individuals as separate subpopulations SP-4, SP-8 and SP-9, respectively. STRUCTURE and BAPS also grouped Nahua and Quechua/Peru individuals as one subpopulation and Asian and South Altaian individuals as another subpopulation. However, ipPCA assigned these individuals to four separate subpopulations consistent with population labels (SP-5, SP-6, SP-11, SP-12).

In order to demonstrate the power of the iterative pruning approach, a comparison was made between the individual subpopulation assignments by ipPCA and clustering from PCA result of the whole dataset (done in a *non-iterative *fashion; last column in Figure 9). The PCA results from Shriver's entire dataset were clustered using the same fuzzy *c*-means clustering algorithm to assign individuals into 12 clusters (the number inferred by ipPCA). The number of clusters to be used for clustering was not calculated independently, e.g., using the Gap statistic since this was not feasible for such a complex dataset. It can be observed that clustering on non-iterative PCA (NI-PCA) results leads to notable differences in individual assignment. For instance, there are three groups with fewer than ten individuals (three Puerto Ricans, eight East Africans, and four New Guineans) and one conglomerate group containing 68 individuals (four Spanish, 11 South Indian Mala, 11 South Indian Brahman, and 42 Caucasians). Furthermore, Asian and South Altaian individuals were assigned together, whereas East African and New Guinea individuals were each separated into two subpopulations.

### Implementation

The ipPCA program was implemented in MATLAB version 2007a. It is available in both graphical user interface (GUI) and MATLAB function. GUI ipPCA is suitable for small datasets, e.g., 50000 markers, while MATLAB function ipPCA can be used for larger datasets. We are currently developing a compiled executable version of this program to broaden the user base. The ipPCA software requires the input data in the following format as a CSV (Comma Separated Value) text file:

Individual identifier, genotype data (0-1-2 encoded) for locus1, genotype data locus2,..., genotype data locus M

The tool for genotype data conversion (STRUCTURE and NCBI dbSNP format supported), the ipPCA MATLAB source codes and instruction for use are available from http://www4a.biotec.or.th/GI/tools/ippca. The outputs from ipPCA in text file format containing individual identifiers, scatter-plot coordinates, and eigenvalues for each iteration are provided.

## Discussion

### Practicality of ipPCA and comparison with other algorithms

We have demonstrated a novel PCA-based analytical framework that accurately resolves population stratification including that of highly structured datasets. With this tool, individuals are assigned to subpopulations and *K *is determined with high accuracy. Moreover, minimal computational effort is required. For example, on a high performance computer (32-core AMD Opteron 2.3 gigahertz with 64 gigabytes of RAM running Linux CentOS operating system), Shriver's dataset requires over three days to compute by STRUCTURE, approximately ten minutes for BAPS while ipPCA needed less than two minutes. Hence, the computational time and the complexity of the datasets were not limitations for ipPCA, unlike other algorithms, e.g., AWclust limits the number of inferred *K *to seven or less.

### Improved clustering through PC selection

The trend observed among datasets analyzed in this study is that early iterations require more PCs for clustering than later ones (Figures 2, 3, 4, 6, and 8). This is in agreement with the findings of the authors of EIGENSTRAT/SmartPCA, who showed that the number of significant eigenvectors (PCs) to resolve structure reflects the number of subpopulations [13,38]. However, the number of *K *subpopulations is not simply defined by the number of significant PCs, since the actual number of PCs needed to reveal subpopulations varies in each case. In ipPCA, the clustering process uses the optimal number of PCs (determined to be the matrix rank), which varies among nested datasets according to the number of individuals at each iteration. After each iteration of ipPCA, the nested datasets have progressively simpler structures so that fewer principal components are used for clustering.

### Inference of *K*

The inference of the number of optimal, or primal, *K *subpopulations and the individual assignment accuracy are critically dependent on each other. High assignment accuracy allows the correct value of *K *to be inferred. The STRUCTURE, BAPS and AWclust algorithms have lower assignment accuracy than ipPCA, and thus their inferences of *K *were found to be incorrect, at least for the simulated data. For real data, STRUCTURE, BAPS and AWclust reported *K *= 3 for the HapMap dataset and could not resolve CHB and JPT as separate subpopulations. The resolution of CHB and JPT subpopulations by ipPCA was consistent with the finding of [39], who identified the subset of informative markers for separating these individuals into two subpopulations.

In order to define subpopulations from the PCA clustering results, ipPCA uses a novel approach which considers only the eigenvalue distribution. By this approach, subpopulations are defined by a standard criterion thus removing subjectivity from the definitions. Rigorous subpopulation definitions are currently lacking, since there is no agreement as to precisely what genetic variation accounts for population structure [40]. The difficulty in defining subpopulations is the main reason why determining *K *is considered very challenging [2,4].

Although ipPCA's inference of *K *appears accurate and robust, there may be situations in which the inferred *K *is incorrect. The proposed ipPCA algorithm would fail to resolve inadequately sampled subpopulations, since the individuals sampled from these subpopulations would be dismissed as PCA outliers, or be assigned to other subpopulations. Conversely, spurious substructure may be reported among subpopulations with very large sampling, although our choice of a very conservative *p*-value for TW statistic in structure detection should militate against this possibility. Data quality is also another issue which could affect inference of *K*; spurious subpopulations may arise from missing data (see algorithm section) and improper sampling of closely related individuals. All of these potential problems are not specific to ipPCA however, and affect any population genetic analysis.

### Assignment accuracy

In terms of individual assignments, there were some notable differences between ipPCA, STRUCTURE, and BAPS for Shriver's dataset. For the optimal number of subpopulations reported by STRUCTURE (*K *= 14) and BAPS (*K *= 5) (see additional files 7 and 6), assignments were inconsistent with what have been reported by other methods [36,41]. From a population evolutionary perspective, some of the assignments made by STRUCTURE and BAPS were not meaningful. For instance, there were two groups that STRUCTURE assigned no individuals to, while five other groups contained fewer than ten individuals (Figure 9). By limiting *K *to twelve as what ipPCA predicted, the assignments made by STRUCTURE changed with removal of the empty groups and amalgamation of south Altaian individuals into one subpopulation (see additional file 8). However, some inconsistencies with accepted patterns of human population structure remained. For example, the subpopulation with pan-African individuals remained. For BAPS, it appears that this algorithm is less sensitive than ipPCA and STRUCTURE, since the assignments revealed individuals with mixed labels (Figure 9). A detailed head-to-head comparison between ipPCA, BAPS, and STRUCTURE was done to demonstrate how our novel non-parametric framework compares against the most recent (BAPS) and well known (STRUCTURE) parametric approaches. Comparisons of ipPCA against other parametric methods (see Background) were not made as they would not be valid, principally because none of these other parametric approaches infer *K *(for review, see [6]). Nonetheless, parametric approaches are still important for certain issues of population genetics not addressed by ipPCA. i.e., determining ancestry proportions.

The ipPCA clustering approach differs from the approaches used by others for clustering PCA results, e.g. Lee et al. [20] who performed clustering on the entire dataset using a large number of PCs. For highly structured populations, this kind of approach is not able to accurately assign individuals to subpopulations, irrespective of the clustering algorithm used (e.g. *k*-means, soft *k*-means, spectral *k*-means, etc. [17]) since the closely related subpopulations are confined in a small region of feature space (see Background section). The greatest problem for the NI-PCA approach is in knowing the number of clusters to be used in clustering. Indeed, the analysis of highly structured datasets in this paper shows that NI-PCA approach fails to accurately assign individuals, even when using the correct *K *inferred by ipPCA (for analysis of simulated data Model 3, see additional file 9). We chose fuzzy *c*-means for clustering since the number of clusters in each ipPCA iteration is restricted to two, hence a simple algorithm is sufficient. Furthermore, the cluster centroids determined by fuzzy *c*-means are more consistent compared with the commonly used *k*-means algorithm [42], which is most important for subpopulation assignment in our case. The clustering algorithm was shown to be highly consistent, as variation in individual assignment owing to variable clustering results from fuzzy *c*-means was observed only for Shriver's dataset.

### Definition of subpopulations according to genetic distance

The iterative pruning approach also defines subpopulations in a manner reflecting the genetic distance, and in some cases the evolutionary path of each subpopulation. As can be seen from the analysis of simulated datasets (Figure 2 and additional file 1) and real datasets in Figures 4, 5, 6, 7, 8, closely related subpopulations are defined after more distantly related ones. This iterative pruning process thus offers a systematic way to define subpopulations according to their degrees of relatedness to one another. By removing the most distant individuals to create nested datasets, one is able to resolve substructure, which would not be revealed otherwise.

For more complex datasets containing subpopulations with individuals of recent admixed origins, these subpopulations were shown by ipPCA as those which contain assigned individuals with different population labels. For instance, two subpopulations were defined from the bovine dataset containing individuals with different breed labels. The overlap of NRC and HOL individuals in a subpopulation is not unexpected, since NRC is not a pure breed and has HOL ancestry. Similarly, the assignment of some SGT individuals to a subpopulation with taurine cattle reflects the fact that SGT is a composite zebu/taurine breed.

For Shriver's dataset, which has a more complex structure than the bovine dataset, twelve subpopulations were defined by ipPCA with individuals assigned consistent with the group labels and the neighbor-joining tree shown in [36]. Four subpopulations appeared to be admixed containing individuals with different labels. Among these four admixed subpopulations, Puerto Rican individuals were assigned to two subpopulations. These assignments reflect the Puerto Rican population history, in which their genomes still contain the signatures of ancestral European and African migrants [36,43]. We are currently developing an admixture extension to ipPCA, which is outside the scope of this paper. In this paper, we wished to provide a solid platform for ipPCA to demonstrate its accuracy in resolving all *K *primal subpopulations, which is crucial for admixture testing.

## Conclusion

We propose some key refinements to the PCA-based algorithm for improved resolution of population genetic stratification. With this new method, individuals can be accurately assigned to subpopulations systematically, thus defining the optimal, or primal, *K *without any assumptions of individuals' origins or degree of relatedness to one another. The power of the technique was demonstrated using datasets with known structure, although we think there is potential to reveal structure in datasets with cryptic stratification.

## Authors' contributions

AI, ST wrote algorithms. AI, PJS, AA, PW, CN, KC, JP, ST analyzed data. AI, PJS, ST wrote the manuscript. All authors read and approved the final manuscript.

## Supplementary Material

Additional file 1 **Figure S1, S2, S3, S4, S5, S6, S7, S8, S9, S10, S11, S12, S13, S14, S15, S16, S17, S18, S19, S20, S21, S22, S23, S24, S25, S26, S27, S28, S29, and S30**. Simulated data Model 3 ipPCA 30 runs results Click here for file

Additional file 2 **Text S1 and S2**. Full list of HapMap SNPs Click here for file

Additional file 3 **Figure S31**. Anomalous clustering result and subpopulation assignment from Shriver's dataset Click here for file

Additional file 4 **Figure S32, S33, and S34**. AWclust results of low complexity datasets Click here for file

Additional file 5 **Text S3, S4, S5, S6, and S7**. STRUCTURE results of low complexity datasets Click here for file

Additional file 6 **Text S8**. BAPS results Click here for file

Additional file 7 **Text S9**. STRUCTURE result of Shriver's dataset with *K *= 14 Click here for file

Additional file 8 **Figure S35**. STRUCTURE result of Shriver's dataset with *K *= 12 Click here for file

Additional file 9 **Figure S36**. Mis-assignment comparison between ipPCA and NI-PCA for Model 3 simulated datasets Click here for file
