# Supplementary figures and images for: Iterative pruning PCA improves resolution of highly structured populations
Source: BMC Bioinformatics. 2009 Nov 23;10:382. doi: 10.1186/1471-2105-10-382 (PMC2790469; doi:10.1186/1471-2105-10-382)

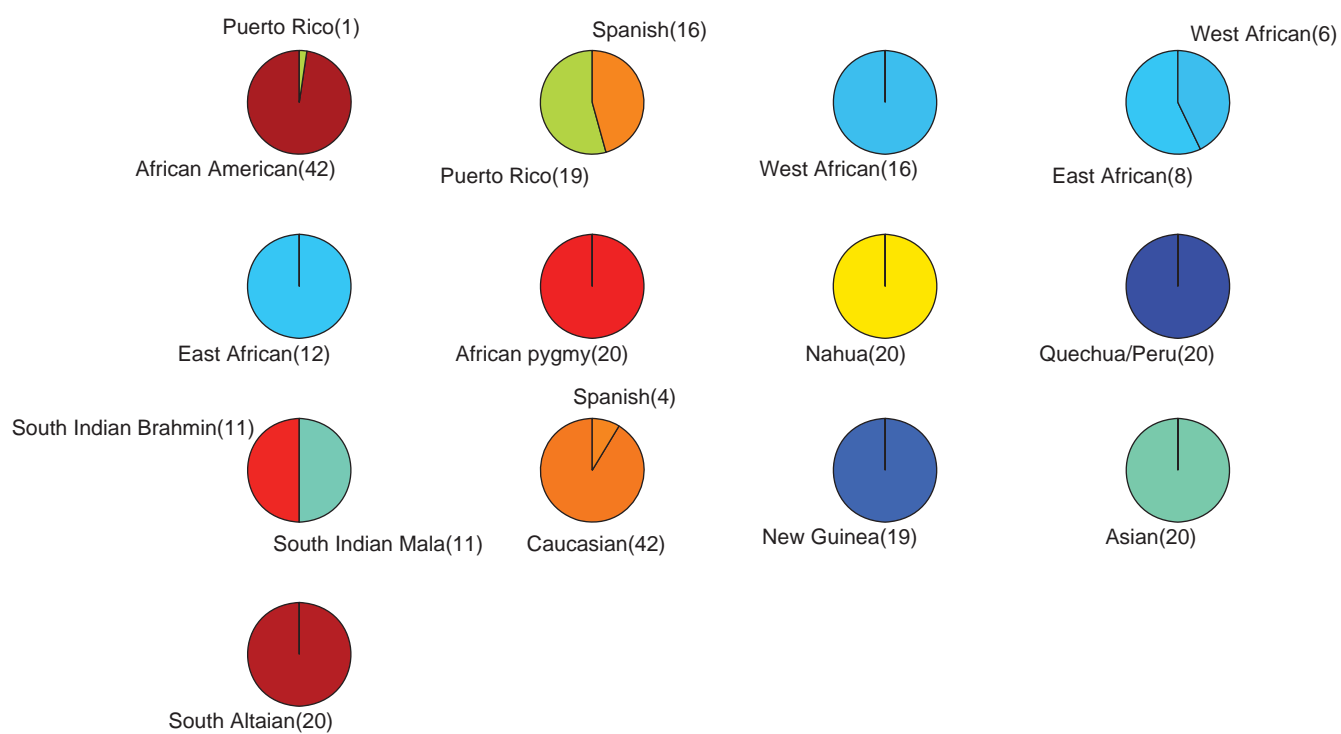

Figure S31. Anomalous clustering result and subpopulation assignment from Shriver's dataset

Supplement: Additional file 3 — Figure S31. Anomalous clustering result and subpopulation assignment from Shriver's dataset [file 1471-2105-10-382-S3.PDF]

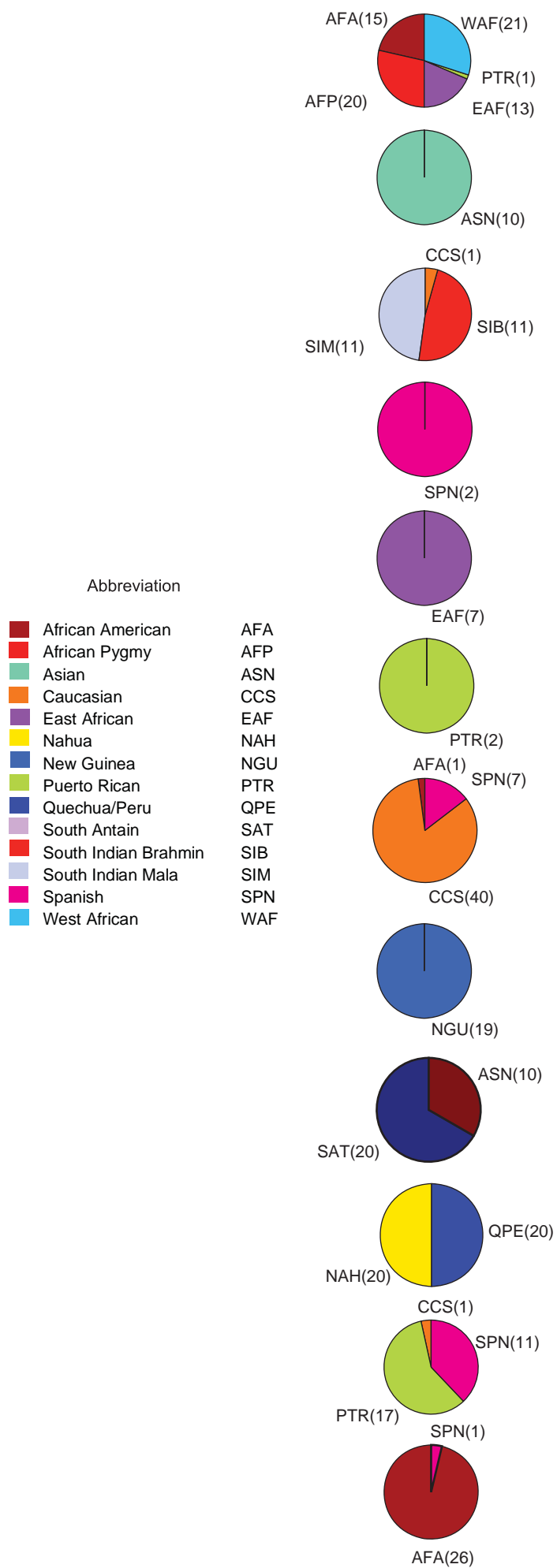

Figure S35. Individual assignment from STRUCTURE with  $K = 12$

Supplement: Additional file 8 — Figure S35. STRUCTURE result of Shriver's dataset with K = 12 [file 1471-2105-10-382-S8.PDF]

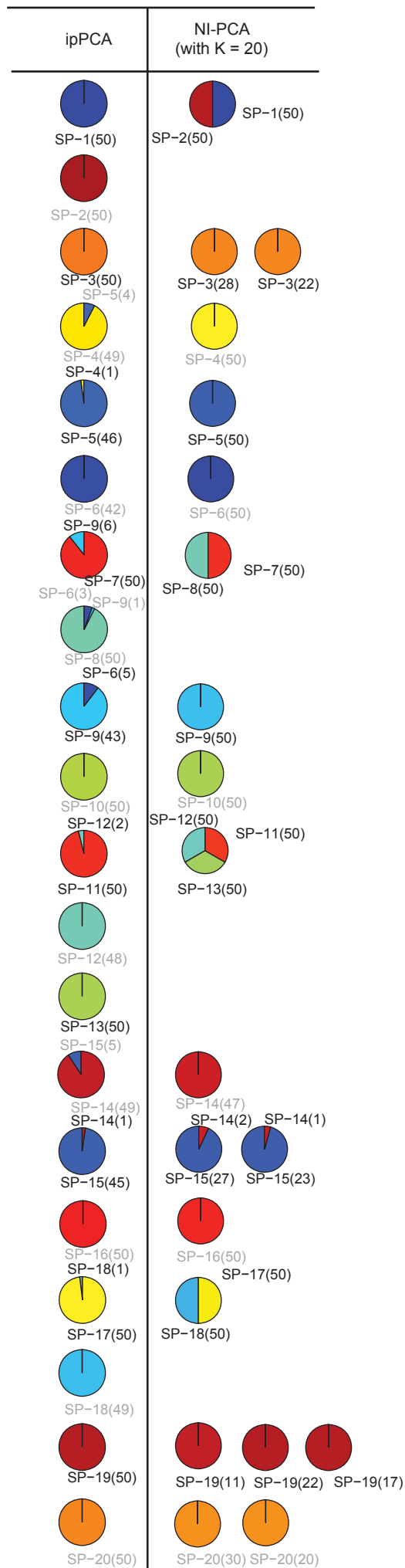

Figure S36 Mis-assignment comparison between ipPCA and NI-PCA for Model 3 simulated datasets

Supplement: Additional file 9 — Figure S36. Mis-assignment comparison between ipPCA and NI-PCA for Model 3 simulated datasets [file 1471-2105-10-382-S9.PDF]
